# Brief inactivation of c-Myc is not sufficient for sustained regression of c-Myc-induced tumours of pancreatic islets and skin epidermis

**DOI:** 10.1186/1741-7007-2-26

**Published:** 2004-12-21

**Authors:** Stella Pelengaris, Sylvie Abouna, Linda Cheung, Vasiliki Ifandi, Sevasti Zervou, Michael Khan

**Affiliations:** 1 Biomedical Research Institute, Department of Biological Sciences, University of Warwick, Gibbet Hill Road, Coventry CV4 7AL, UK

## Abstract

**Background:**

Tumour regression observed in many conditional mouse models following oncogene inactivation provides the impetus to develop, and a platform to preclinically evaluate, novel therapeutics to inactivate specific oncogenes. Inactivating single oncogenes, such as c-Myc, can reverse even advanced tumours. Intriguingly, transient c-Myc inactivation proved sufficient for sustained osteosarcoma regression; the resulting osteocyte differentiation potentially explaining loss of c-Myc's oncogenic properties. But would this apply to other tumours?

**Results:**

We show that brief inactivation of c-Myc does not sustain tumour regression in two distinct tissue types; tumour cells in pancreatic islets and skin epidermis continue to avoid apoptosis after c-Myc reactivation, by virtue of Bcl-x_L _over-expression or a favourable microenvironment, respectively. Moreover, tumours progress despite reacquiring a differentiated phenotype and partial loss of vasculature during c-Myc inactivation. Interestingly, reactivating c-Myc in β-cell tumours appears to result not only in further growth of the tumour, but also re-expansion of the accompanying angiogenesis and more pronounced β-cell invasion (adenocarcinoma).

**Conclusions:**

Given that transient c-Myc inactivation could under some circumstances produce sustained tumour regression, the possible application of this potentially less toxic strategy in treating other tumours has been suggested. We show that brief inactivation of c-Myc fails to sustain tumour regression in two distinct models of tumourigenesis: pancreatic islets and skin epidermis. These findings challenge the potential for cancer therapies aimed at transient oncogene inactivation, at least under those circumstances where tumour cell differentiation and alteration of epigenetic context fail to reinstate apoptosis. Together, these results suggest that treatment schedules will need to be informed by knowledge of the molecular basis and environmental context of any given cancer.

## Background

Various mouse models of tumourigenesis have been established using conditional systems to either induce or knockout particular genes (oncogenes and tumour suppressors, respectively) in a tissue-specific and time-dependent manner. The ability to switch expression of a given oncogene 'on' or 'off' *in vivo *has provided insight into the mechanisms by which certain oncogenes can initiate tumourigenesis either alone or in combination with other genetic lesions, and importantly, whether inactivation of the initiating oncogene is sufficient to cause tumour regression (reviewed in: [1,2]). Given the importance of oncogene activation in human cancers, specific targeting of oncogenic pathways provides a potentially effective therapeutic strategy. For example, targeting of the HER2/Neu receptor tyrosine kinase (which is overexpressed in up to 30% of primary human breast cancers) with the neutralizing antibody Trastuzumab has been used successfully in clinical trials, in combination with other agents, to slow disease progression (refs in [3]). Similarly, patients with chronic myelogenous leukaemia (CML) have been effectively treated with the ABL kinase inhibitor, Imatinib (Gleevec), inducing clinical remission whilst in the CML phase [4,5].

Several studies using conditional mouse models of various cancers have unexpectedly shown that inactivation of the initiating oncogene is sufficient for reversal not only of the primary tumour but also of invasive and metastatic lesions, many of which contain multiple genetic and epigenetic alterations [6-18].

The tumour regression observed in many of these models following sustained oncogene inactivation provides a powerful platform on which to build a deeper understanding of fundamental tumour biology and with which to preclinically evaluate novel therapeutics to target specific genes. A recent study has shown that brief inactivation (10 days) of c-Myc was sufficient for the sustained regression of c-Myc induced invasive osteogenic sarcomas in transgenic mice [19]; subsequent re-activation of c-Myc led to extensive apoptosis rather than restoration of the neoplastic phenotype. Possible explanations for this outcome include changes in epigenetic context that may have occurred within the cell type, that is, presumably between the immature cell in which c-Myc was originally activated and the more differentiated cell resulting from subsequent (brief) inactivation of c-Myc. In this tumour model, although c-Myc expression is initiated in immature osteoblasts during embryogenesis, subsequent inactivation of c-Myc in osteogenic sarcoma cells induces differentiation into mature osteocytes. Therefore, re-activation of c-Myc now takes place in a different cellular context and induces apoptosis rather than neoplastic progression. However, irrespective of the actual underlying mechanisms, these intriguing findings suggest the novel possibility of employing transient inactivation of c-Myc as a therapeutic strategy in certain cancers, thus limiting potential toxic effects resulting from prolonged therapeutic inactivation [1,2]. In fact, there is widespread interest in determining the optimal timing of existing therapies, including trials of pulsatile or 'metronomic' chemotherapy regimens in various cancers. Self-evidently, therefore, it was essential to determine if this phenomenon was unique to this mouse model or if sustained regression of tumours originating in different tissues and under differing circumstances could also be induced by transient c-Myc inactivation.

Previously, we have shown that sustained c-Myc inactivation in locally invasive pancreatic islet tumours (induced by c-Myc activation in β-cells on a background of Bcl-x_L _overexpression) induced β-cell growth arrest and re-differentiation into mature β-cells, accompanied by the collapse of tumour vasculature and tumour cell mass resulting from apoptosis, despite the constitutive expression of Bcl-x_L _in the tumour cells [9]. Similarly, in pre-malignant skin epidermal tumours (papillomatosis) induced following activation of c-Myc alone, skin lesions completely regressed within 4 weeks after sustained inactivation of c-Myc [8]. However, given the continual outward migration and shedding of growth-arrested and re-differentiated keratinocytes from the skin surface, it was not established whether this action alone was responsible for the removal of skin tumour cells or if apoptosis also played a part.

These conditional models, in which c-Myc-induced tumours of pancreatic islets and skin epidermis can be initiated at any given time in the adult animal, were chosen for the studies presented here and offer several advantages (reviewed in [20]). First, tumourigenesis after c-Myc activation is initiated and proceeds by different routes, with inherent pro-apoptotic activity avoided by an additional genetic alteration (expression of the anti-apoptotic protein, Bclx_L_) as is the case in the islet tumourigenesis model, or by the presence of survival cues in the microenvironment, as seen in c-Myc-induced skin tumours. Second, we already know that sustained c-Myc inactivation leads to regression in both cases.

Here we show the consequences of inactivating c-Myc transiently, for a period of 4 to 9 days, in these distinct tumour types *in vivo*. In contrast to the osteogenic sarcoma model, re-activating c-Myc in islet tumours does not lead to accelerated β-cell apoptosis, but rather restores the oncogenic properties of c-Myc, rapidly re-initiating β-cell proliferation, loss of differentiation, loss of E-cadherin, local invasion and angiogenesis. This occurs despite the re-differentiation of previously c-Myc-activated tumour cells to a more mature phenotype and the loss of some of the newly acquired vasculature, occurring during the period of c-Myc inactivation. Moreover, as no new β-cells arise during the period of c-Myc inactivation, replication is probably restored in those same cells that have previously experienced c-Myc activation. Similarly, in epidermis, reactivating c-Myc in suprabasal keratinocytes does not result in apoptosis, which remains confined to the shedding areas of parakeratosis at the skin surface, but restores the papillomatous phenotype, inducing cell proliferation and dysplasia. These results are in line with a very recent study in a different system, published while this manuscript was under consideration. Shachaf and colleagues demonstrated that invasive c-Myc-induced hepatocellular carcinomas regress when *c-Myc *expression is turned off but, interestingly, some tumour cells remain 'dormant' even for prolonged periods and contribute to cancer progression if *c-Myc *expression is subsequently reinitiated [21].

Taken together, these findings suggest that a cautious approach is required in considering cancer therapies aimed at transient oncogene inactivation. First, a more comprehensive understanding of the genetic basis and environmental context of any individual tumour would be required in order to predict the likely success of such a treatment schedule. Second, at least under those circumstances where tumour cell differentiation and alteration of epigenetic context would not be predicted to reinstate apoptosis and no alternative mechanism exists for tumour cell removal, sustained inactivation of the offending oncogene would seem the desired therapeutic goal.

## Results

### Brief inactivation of c-Myc in islet tumours does not sustain tumour regression

Islet tumours were induced in nine mice by activation of c-MycER^TAM ^(c-Myc) in β-cells of adult *pIns-c-MycER*^*TAM *^mice cross-bred with *RIP7-Bcl-x*_*L *_mice [22] by daily intraperitoneal (IP) administration of 4-OHT for 14 days as previously described [9]. Three mice from different litters were sacrificed for histological examination of the pancreas. c-Myc was then inactivated by 4-OHT withdrawal (see Methods) for 9 days in the remaining six littermates (two from each litter), after which time one mouse from each litter was sacrificed for collection of pancreata and analysis. In the remaining three mice, reactivation of c-Myc by daily IP injection of 4-OHT was carried out for 5 days before sacrifice and analysis. For each analysis, sections (5–10μm) were cut throughout the length of the pancreas and every tenth section was selected for histological and immunohistochemical examination.

**Figure 1:**
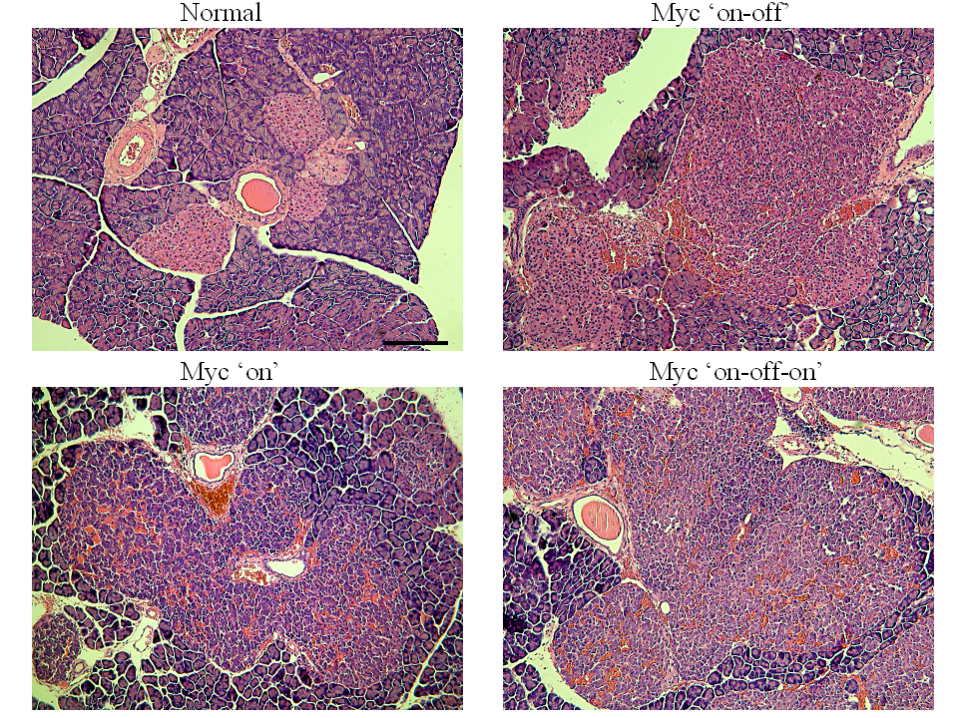
**Histological comparison of pancreatic islets from *pIns-c-MycER*^*TAM*^/*Bcl-x*_*L *_transgenic mice following activation, inactivation and reactivation of c-MycER^TAM ^(c-Myc)**. Paraffin-embedded fixed sections of pancreata from *c-MycER*^*TAM*^/*Bcl-x*_*L *_mice were examined histologically by H&E staining. In the absence of 4-OHT (inactive c-Myc) islets exhibited normal morphology (**Normal**). Activation of c-Myc for 14 days following daily IP injection with 4-OHT induced the formation of hyperplastic, angiogenic islets (**c-Myc on**). Islets following subsequent inactivation of c-Myc for 9 days by withdrawing 4-OHT show evidence of vasculature collapse (**c-Myc on-off**). Reactivation of c-Myc for 5 days in partially regressed islets restored the neoplastic phenotype (**c-Myc on-off-on**). Scale bar: 100 μm

**Figure 2:**
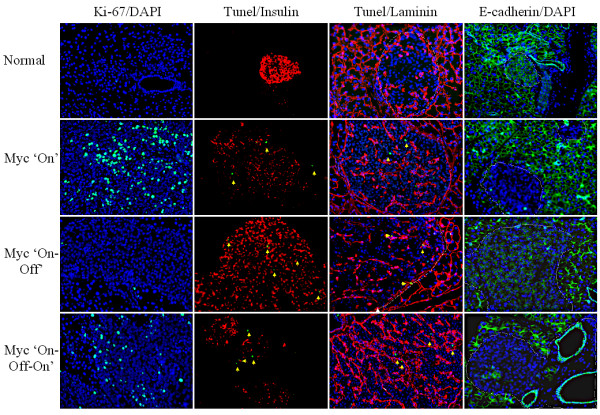
**Immunohistochemical comparison of pancreatic islets from *pIns-c-MycER*^*TAM*^/*Bcl-x*_*L *_transgenic mice following activation, inactivation and reactivation of c-MycER^TAM ^(c-Myc)**. Frozen or paraffin-embedded fixed sections of pancreata from *c-MycER*^*TAM*^/*Bcl-x*_*L *_mice were examined immunohistochemically for the detection of: β-cell proliferation using anti-Ki-67 antibodies; apoptosis of β-cells or vascular endothelial cells (anti-insulin (red) or anti-laminin (red) antibodies together with TUNEL (green). Apoptotic nuclei are highlighted with arrows; loss of cell-cell contact (anti-E-cadherin antibodies). In the absence of 4-OHT (inactive c-Myc) less than 0.1% of β-cells are proliferating or undergoing apoptosis (**Normal**). Islets from *c-MycER*^*TAM*^/*Bcl-x*_*L *_mice following activation of c-Myc for 14 days showed a substantial degree of β-cell proliferation concomitant with down-regulation of insulin immuno-staining (loss of differentiation) and loss of cell-cell contact (**c-Myc on**). Some apoptotic β-cells were detected but no apoptotic cells were present in the vascular endothelium. Inactivation of c-Myc for 9 days led to cessation of β-cell proliferation, up-regulation of insulin and E-cadherin with some vascular endothelial cell apoptosis (**c-Myc on-off**). Reactivation of c-Myc for 5 days induced β-cell proliferation, down-regulation of insulin and E-cadherin (**c-Myc on-off-on**). Importantly, the same degree of apoptosis as that seen in "c-Myc on" tumours was evident. General nuclear stain: Dapi. Scale bar: 100 μm

In the absence of 4-OHT, *pIns-c-MycER*^*TAM*^/*RIP-Bcl-x*_*L *_double transgenic mice exhibited normal islet morphology (Figure 1) with no measurable β-cell proliferation or apoptosis (Figure 2). As expected, activation of c-Myc for 14 days triggered the progression of angiogenic islet tumours (Figure 1) accompanied by β-cell proliferation, loss of differentiation (as demonstrated by down-regulation of insulin) and down-regulation of the intercellular adhesion molecule E-cadherin (Figure 2). Following inactivation of c-Myc for 9 days, histological analyses of pancreata showed signs of vasculature collapse particularly in larger islets (Figure 1-extravasated erythrocytes), accompanied by cessation of β-cell proliferation, re-differentiation (up-regulation of insulin) and reestablishment of cell-cell contacts as E-cadherin expression was restored (Figure 2). Vascular endothelial cell apoptosis was detected in larger islets by co-immunostaining of vascular basal lamina with anti-laminin antibodies together with TUNEL (Figure 2), with only a small number of apoptotic β-cells detected at this time-point (average of 1 cell per islet section) following co-immunostaining of β-cells with anti-insulin antibodies together with TUNEL (Figure 2).

Reactivation of c-Myc for 5 days in partially regressed islet tumours (9 days of inactive c-MycER^TAM^) led to restoration of the oncogenic properties of c-Myc (Figure 1 and 2): β-cell proliferation, loss of differentiation (down-regulation of insulin), and loss of cell-cell contacts (down-regulation of E-cadherin). In contrast to the mouse model of osteogenic sarcoma [19], where reactivation of c-Myc in growth-arrested, re-differentiated osteocytes induced their rapid demise through apoptosis, reactivation of c-Myc in islet tumours did not lead to an increase in the number of cells undergoing apoptosis compared to the original islet tumours formed after 14 days of c-Myc activation (Figure 2). Importantly, in contrast to partially regressed islet tumours (9 days of c-Myc inactivation), where most apoptotic cells were found in the vasculature (Figure 2), the majority of apoptotic cells in both the original 14-day islet tumours and reactivated tumours were not present within the vasculature but rather found predominantly adjacent to blood vessels (averages of 3–4 cells per islet section c-Myc 'on' versus 3–5 cells per islet c-Myc 'on-off-on', both representing less than 0.1% of islet cells). A small number of these TUNEL-positive cells could be identified as β-cells, as deduced following co-staining with insulin (Figure 2) or Nkx6.1 (data not shown). Of the remaining apoptotic cells, some could be identified as leukocytes by co-staining with CD45 (data not shown). The identity of the remainder is unclear, but could include some β-cells with complete loss of normal differentiation markers. Despite the presence of apoptotic cells during c-Myc activation, levels are clearly insufficient to prevent islet tumour progression as c-Myc-induced β-cell proliferation far exceeds apoptosis and tumours rapidly and inexorably expand over time.

**Figure 3:**
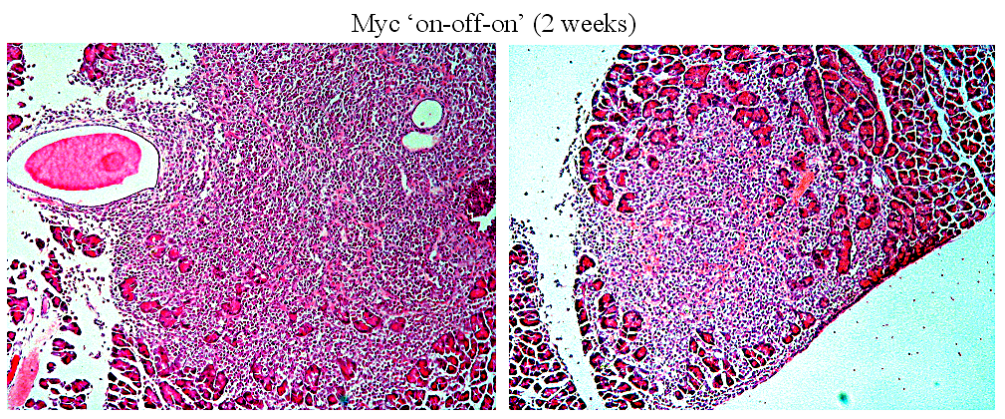
**Pancreatic islets from two *pIns-c-MycER*^*TAM*^/*Bcl-x*_*L *_transgenic mice following activation, inactivation, and then reactivation of c-MycER^TAM ^(c-Myc)**. Paraffin-embedded fixed sections of pancreata from *c-MycER*^*TAM*^/*Bcl-x*_*L *_mice were examined histologically by H&E staining. Activation of c-Myc for 14 days by daily IP injection with 4-OHT was followed by subsequent inactivation of c-Myc for 9 days by withdrawing 4-OHT, and then reactivation of c-Myc for 14 days results in further progression of the neoplastic phenotype -**c-Myc on-off-on (2 weeks)**. Extending the period of c-Myc reactivation results in further expansion of the tumour mass, marked dysplasia and invasion.

In order to establish whether islet tumour progression was indeed being maintained following reactivation of c-Myc we examined a longer period of c-Myc reactivation. In this case, mice were treated daily with IP 4-OHT for 14 days (following an initial 2 week period of c-Myc activation and 9 day transient period of c-Myc inactivation). Sections were cut throughout the pancreas and selected sections at every 100 μm were stained with H&E for histological analysis as well as with immunohistochemical markers for proliferation (Ki-67), differentiation (insulin), apoptosis (TUNEL) and loss of cell-cell contact (E-cadherin). Importantly, pancreatic islets following 14 days of c-Myc reactivation showed no signs of tumour regression but rather progression of islet tumourigenesis, with the vast majority of islets showing more pronounced invasion (adenocarcinoma) (Figure 3).

### Brief inactivation of c-Myc in papillomatous lesions of the skin does not sustain tumour regression

Development of papillomatosis (a pre-malignant lesion of skin epidermis resembling actinic keratosis in humans) was induced in nine mice following activation of c-MycER^TAM ^(c-Myc) in suprabasal keratinocytes of adult *Involucrin-c-MycER*^*TAM *^transgenic skin by daily topical administration of 4-OHT for 14 days as previously described [8]. Three mice from different litters were sacrificed for histological examination of the skin. c-Myc was then inactivated by 4-OHT withdrawal (see Methods) for 5 days in the remaining six littermates (two from each litter), after which time one mouse from each litter was sacrificed for collection of skin and analysis. In the remaining three mice, reactivation of c-Myc by daily topical application of 4-OHT was carried out for 5 days before sacrifice and analysis. We chose to inactivate c-Myc for 5 days in order to minimize the loss of neoplastic keratinocytes through outward migration and shedding from the skin surface, prior to reactivation of c-Myc, in order to maximise potential detection of any keratinocyte apoptosis. For each analysis, sections (5–10μm) were cut throughout two 10 mm pieces of skin and every tenth section was selected for histological and immunohistochemical examination.

**Figure 4:**
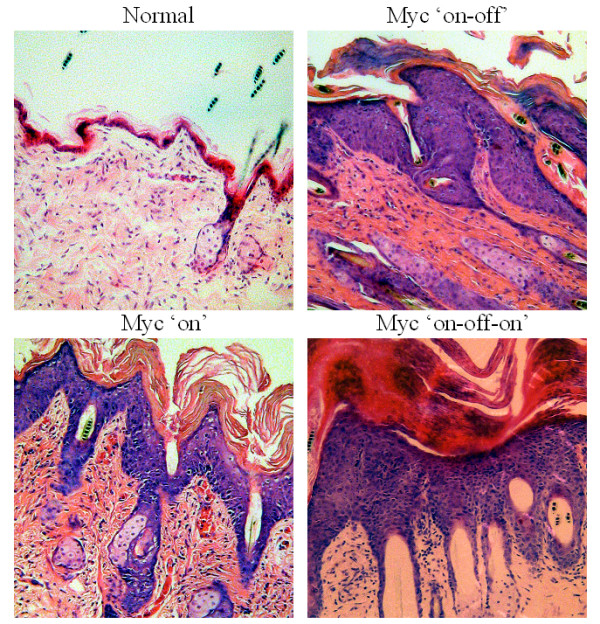
**Histological comparison of skin epidermis from *Involucrin-c-MycER*^*TAM *^transgenic mice following activation, inactivation, and reactivation of c-MycER^TAM ^(c-Myc)**. Paraffin-embedded fixed sections of skin from *Involucrin-c-MycER*^*TAM *^mice were examined histologically by H&E staining. In the absence of 4-OHT (inactive c-Myc) the epidermis shows normal histology (**Normal**). Activation of c-Myc in skin treated topically with 4-OHT daily for 14 days induced hyperplasia, dysplasia and angiogenesis within papillomatous lesions (**c-Myc on**). Subsequent inactivation of c-Myc for 5 days following 4-OHT withdrawal led to redifferentiation of suprabasal keratinocytes as evidenced by the appearance of the granular cells and loss of dysplasia (**c-Myc on-off**). Reactivation of c-Myc for 5 days restored the papillomatous phenotype (**c-Myc on-off-on**). Scale bar: 100 μm

**Figure 5:**
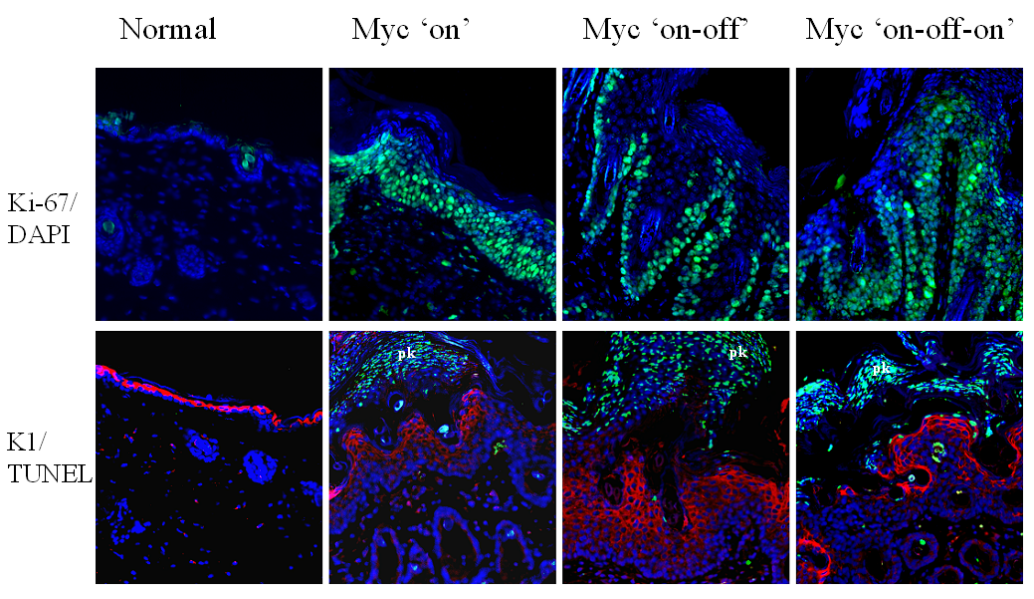
**Immunohistochemical comparison of skin epidermis from *Involucrin-c-MycER*^*TAM *^transgenic mice following activation, inactivation, and reactivation of c-MycER^TAM ^(c-Myc)**. Frozen or paraffin-embedded fixed sections of skin from *Involucrin-c-MycER*^*TAM *^mice were examined immunohistochemically for proliferation using anti-Ki-67 antibodies, and apoptosis of suprabasal keratinocytes using anti-keratin 1 antibodies (red) together with TUNEL (green). In the absence of 4-OHT (inactive c-Myc) there were no proliferating or apoptotic keratinocytes within the suprabasal layer (**Normal**). Activation of c-Myc in skin treated topically with 4-OHT daily for 14 days induced proliferation of a substantial proportion of suprabasal keratinocytes (**c-Myc on**). The only detectable apoptotic cells were confined to shedding keratinocytes present at the surface and in nucleated cornified layers (parakeratosis). Inactivation of c-Myc for 5 days led to a reduction in the proportion proliferating suprabasal keratinocytes and no increase in the presence of apoptotic cells (**c-Myc on-off**). Reactivation of c-Myc for 5 days restored the tumour phenotype by inducing proliferation of suprabasal keratinocytes in the absence of any further apoptosis, TUNEL-positive cells again confined to shedding keratinocytes present at the surface and in nucleated cornified layers (**c-Myc on-off-on**). General nuclear stain: Dapi. Scale bar: 100 μm

In the absence of 4-OHT, *Involucrin-c-MycER*^*TAM *^transgenic mice exhibited normal skin morphology (Figure 4) with no detectable suprabasal keratinocyte proliferation or apoptosis (Figure 5). As expected, activation of c-Myc for 14 days triggered the progression of papillomatosis with marked epidermal hyperplasia, dysplasia, angiogenesis, and formation of nucleated cornified layers (parakeratosis) (Figure 4). These "parakeratotic tiers" gave the appearance of dry, scabby lesions, which were eventually lost through lifting or flaking from the surface. The hyperplastic phenotype resulted from c-Myc induced proliferation of suprabasal keratinocytes, as detected using antibodies specific for the proliferation marker Ki-67 (Figure 5). As demonstrated in our earlier studies [8], c-Myc alone in the absence of any ectopic anti-apoptotic lesion is sufficient to induce pre-malignant neoplastic lesions in the skin. This contrasts with results in the pancreatic islet β-cells, where c-Myc activation alone induces widespread apoptosis [9]. Activation of c-Myc in the epidermis, however, is associated with essentially no detectable apoptosis when examined by co-staining with the specific suprabasal keratinocyte marker keratin 1 together with TUNEL (Figure 5). The only detectable TUNEL positive cells were those present at the surface of the skin about to be shed and nucleated cells within the parakeratotic tiers (Figure 5).

Subsequent inactivation of c-Myc for 5 days led to redifferentiation of suprabasal keratinocytes as evidenced by the appearance of granular cells and loss of dysplasia (Figure 4) concomitant with a marked reduction in the number of proliferating suprabasal keratinocytes (Figure 5). Importantly, there was no increase in the number of cells undergoing apoptosis, with TUNEL positive cells again confined to shedding keratinocytes (Figure 5), indicating that regression of these skin lesions occurs through loss of neoplastic keratinocytes by shedding. Although tumour vasculature was examined for endothelial apoptosis, there was no measurable increase in cell death at this particular time-point. It is likely, however, that a more extensive analysis would determine the particular stages at which vascular collapse occurs following c-Myc inactivation.

Reactivation of c-Myc in skin lesions for 5 days led to restoration of papillomatosis (Figure 4). In contrast to the osteogenic sarcoma model, reactivation of c-Myc in growth arrested, redifferentiated keratinocytes did not result in increased apoptosis, despite the absence of an anti-apoptotic lesion (Figure 5), but again resulted in increased levels of suprabasal proliferation extending beyond the basal compartment (Figure 5); but as in the c-Myc 'on', no replicating cells were present in the granular layer. However, we cannot confirm what proportion of replicating suprabasal keratinocytes, following c-Myc reactivation, had previously experienced c-Myc activation. This question arises as new 'c-Myc naïve' keratinocytes are likely to have entered the suprabasal compartment as a result of on-going basal layer replication during the transient period of c-Myc inactivation.

## Discussion

The potential to inactivate or mitigate the action of oncogenes is increasingly being exploited in the design of new therapeutic agents to reverse tumourigenesis in cancer therapy [1,23-25]. Importantly, despite the seeming genetic complexity of many cancers, a compelling body of evidence suggests that inactivation of single oncogenes can be sufficient for tumour regression. In several transgenic mouse models of cancer, the generation of invasive/metastatic tumours in which more than one genetic event is involved can be reversed following inactivation of a single oncogene [7,9,12,16]. In addition, c-Myc-induced lymphomas shown to be genomically complex and unstable regressed following c-Myc inactivation [15]. Strikingly, extensive lung metastases, arising in mice bearing *Neu*-induced mammary tumours, rapidly and fully regressed following inactivation of *Neu*, despite tumour cells acquiring additional mutations [14]. These findings suggest that some metastatic lesions may remain responsive to therapeutic intervention originally targeted to the primary lesion, although some tumours have been shown to escape dependence upon the initiating oncogene [6,7,11,14,16,19].

Recent data from a mouse model of osteogenic sarcoma showed that even transient c-Myc inactivation can result in sustained tumour regression [19]. In this model, reactivation of c-Myc after a brief period of tumour regression led to extensive apoptosis of osteoblasts rather than restoration of the tumour phenotype. This increased sensitivity to apoptosis may be due to epigenetic changes that have occurred within the newly differentiated cells (when c-Myc was inactivated). In light of these findings, it was important to establish whether reactivating c-Myc in other tumour models, consisting of other cell types, would also lead to apoptosis. In other words, would such cells behave differently from the original differentiated cell (in which c-Myc was first activated) and become more sensitive to Myc-induced apoptosis as a result of epigenetic changes?

Here we show, in contrast to the osteogenic sarcoma model, that re-activating c-Myc in islet β-cell tumours restores the oncogenic properties of c-Myc, rapidly re-initiating β-cell proliferation, loss of differentiation, loss of E-cadherin, local invasion and angiogenesis. This occurs despite the re-differentiation of previously c-Myc-activated tumour cells to a more mature phenotype and the loss of some of the newly acquired vasculature, occurring during the period of c-Myc inactivation. Similarly, in epidermis, reactivating c-Myc in suprabasal keratinocytes does not result in apoptosis, which remains confined to the shedding areas of parakeratosis at the skin surface, but restores the papillomatous phenotype, inducing cell proliferation and dysplasia.

Self-evidently, to restore vulnerability to the pro-apoptotic activity of c-Myc would necessitate the tumour cells losing their resistance to apoptosis. In this case the results of Jain et al. [19] may be explained by the origin of osteosarcomas in their model in bone progenitor cells, which we assume were able to avoid c-Myc-induced apoptosis. Subsequently, despite the retention of some features of immaturity, these cells progress to a differentiated phenotype upon transient c-Myc inactivation, which renders them susceptible to apoptosis when c-Myc is reactivated. The assumption we must make here is that whatever uncharacterised additional mutations these cells may have acquired along their journey to malignancy, these did not include mutations able to confer resistance to apoptosis in the more mature osteocyte. One may speculate about the underlying mechanisms, one possibility being the lack of a selective evolutionary advantage to acquisition of an anti-apoptotic lesion, given that some of these cells must already have been capable of avoiding apoptosis – perhaps due to their primitive developmental stage.

In our *pIns-MycER*^*TAM *^mice, where mature β-cells are normally highly sensitive to the pro-apoptotic activity of c-Myc [9], invasive β-cell tumours only originate once apoptosis is prevented, in this case by constitutive over-expression of the protein Bclx_L_. Without such apoptosis suppression the majority of β-cells undergo apoptosis upon c-Myc activation, rendering mice diabetic within a few days. This may be one difference between our own results and those of Jain et al. [19], namely that in our system an acquired resistance to apoptosis is required from the outset, but once this is in place and stays in place, tumours develop and progress whatever the differentiation status of these cells. In contrast, in the system studied by Jain et al. [19], cells can lose their resistance to apoptosis whilst c-Myc is inactivated. This notion is supported by the rapid continuation of replication and tumour growth in our mice after a transient period of c-Myc inactivation despite this being sufficient to restore a differentiated phenotype in β-cells. In contrast to the osteogenic sarcoma model [19], it is less likely that epigenetic changes have occurred: c-Myc is originally activated in mature islet β-cells of the adult pancreas, and following inactivation of c-Myc in islet tumours there is a restoration to differentiated adult β-cells. Importantly, despite the beginning of vascular collapse during c-Myc inactivation, reactivating c-Myc appears to result not only in further growth of the tumour, but also re-expansion of the accompanying angiogenesis and more pronounced islet invasion (adenocarcinoma). The persistence of β-cell resistance to the pro-apoptotic activity of c-Myc is probably conferred by the continued presence of the anti-apoptotic protein Bclx_L_.

Although the general consensus view is that human cancers largely arise from stem cells or precursor cells, it is equally plausible that some cancers might also originate from more mature cells. Intriguingly, recent studies in pancreatic islets of the adult mouse suggest that putative stem cells do not proliferate and produce new β-cells in adult animals, but rather β-cell turnover is maintained by proliferation of mature β-cells [26]. It is therefore quite plausible that islet tumours might also originate from these more mature cells, which as the major source of replicating cells in the adult would also be those most likely to acquire cancer-causing mutations. Another illuminating study from Bachoo et al. [27] shows that dysregulation of specific genetic pathways, rather than cell-of-origin, dictates the emergence and phenotype of high-grade gliomas.

In order to investigate whether the absence of an anti-apoptotic mutation would result in a shift from resistance to apoptosis towards vulnerability following transient inactivation of c-Myc, we examined a different mouse tumour model. In suprabasal keratinocytes, c-Myc activation induces relentless replication leading to hyperplasia, angiogenesis and a premalignant phenotype resembling actinic keratosis. In this system, differentiated suprabasal keratinocytes presumably avoid apoptosis due to the permissive environment of the epidermis, as they readily undergo apoptosis when removed from this environment [8]. It was, however, possible that the dramatic increase in cell numbers following c-Myc-induced epidermal hyperplasia might overwhelm the survival signals within this tissue, which although sufficient to prevent any discernible apoptosis during sustained c-Myc activation might no longer be able to prevent it after a transient inactivation (with any accompanying restoration of normal differentiation). In fact, we see no obvious change in the behaviour of keratinocytes from before to after a period of c-Myc inactivation. Apoptosis remains confined to the area of parakeratosis, which accompanies c-Myc-induced hyperplasia and papillomatosis. At these time-points we do not see any prominent endothelial cell apoptosis, so the exact point at which the angiogenesis collapses is not known.

Interestingly, a recent publication suggests that following a transient period of c-Myc inactivation, at least some previously c-Myc activated suprabasal keratinocytes may differentiate to an extent sufficient to render them unable to re-enter the cell cycle after c-Myc reactivation [28]. This is supported by our original work suggesting that more differentiated suprabasal keratinocytes in the granular compartment are generally refractory to c-Myc-induced replication [8]. In this case, restoration of papillomatous lesions in *Involucrin-c-MycER*^*TAM *^mice might instead result largely from the replication of 'c-Myc naïve' keratinocytes newly generated from the basal layer during the period of c-Myc inactivation [28]. Whatever the underlying explanation, it is difficult to be sure that all previously c-Myc activated cells have undergone irreversible growth arrest and therefore make no contribution to restoration of the tumour phenotype. This issue may only be resolved fully by the development of a suitable labelling technique, which could indelibly mark all c-Myc activated keratinocytes, but only up to the point at which c-Myc is inactivated and not when c-Myc is reactivated.

Fortunately, with the islet model, there is no such element of doubt. In this case no new β-cells are formed during the period of c-Myc inactivation, with replication essentially absent within the pancreas during this period. The replication of β-cells after c-Myc reactivation must, therefore, be taking place in those same cells that have re-differentiated whilst c-Myc was deactivated. Therefore, one can confidently state that transient c-Myc inactivation in tumours originating in *pIns-c-MycER*^*TAM*^/*RIP7-Bcl-x*_*L *_double transgenic mice will not lead to either apoptosis or irreversible growth arrest in tumour cells. In a recently published study, Shachaf and colleagues demonstrate in a Tet-regulatable conditional mouse model that invasive c-Myc-induced hepatocellular carcinomas regress when ectopic *c-Myc *expression is turned off. Importantly, by employing a bioluminescence technique to label hepatocellular cancer cells, it was shown that some erstwhile tumour cells re-differentiate but avoid apoptosis and remain 'dormant' even for prolonged periods after *c-Myc *transgene expression is turned off. These labelled cells can then once more contribute to cancer progression if *c-Myc *transgene expression is subsequently restored [21].

Extrapolating from these various results one may assume that where avoidance of c-Myc induced apoptosis is a product of cellular immaturity (as may be the case in some stem cell populations), then as long as c-Myc inactivation induces differentiation, and, presumably, no anti-apoptotic mutation has been acquired, a transient period may suffice for sustained tumour regression. However, in many cases where an anti-apoptotic lesion is also present (loss of p53/p19ARF; upregulation of antiapoptotic Bcl2 family members etc), or potentially the microenvironment continues to prevent apoptosis, sustained inactivation would be essential for tumour regression. Moreover, it can also be stated that partial reversal of angiogenesis, at least in the islet tumours, will not have any lasting impact on tumour progression if angiogenesis automatically continues apace of further growth of the tumour. Finally, it seems likely that reacquisition of a differentiated phenotype does not preclude previously c-Myc activated cancer cells from re-exhibiting cancer behaviour once c-Myc is reactivated – removal of these cells by apoptosis or other means would seem necessary to remove the threat of cancer recrudescence.

Identifying these key differences in behaviour between different cell types/developmental stages is not an academic exercise, but can give vital information about the mechanisms and context whereby oncogene activity may be determined, which in addition to the biological interest might also provide new knowledge of direct relevance to human cancer.

Given the fact that deregulation of c-Myc expression is one of the most frequently described abnormalities in human cancers and has been observed in β-cell derived tumours and in human skin epidermal tumours [29-32], our observations may have important ramifications for human cancers. It seems likely that therapies directed at oncogene targets will need to be individually tailored to fit the individual tumour types. Thus, detailed knowledge of the molecular 'road map' to cancer for any individual tumour would be needed before determining the optimal treatment targets and therapeutic schedule. In some cases, where describing the molecular basis of the tumour suggests no inherent resistance to apoptosis, transient c-Myc inactivation may prove an effective part of the therapeutic strategy, whereas identifying the presence of lesions known to suppress c-Myc apoptosis would direct therapy at maintaining sustained c-Myc inactivation. Moreover, such detailed molecular information on the cancer cells would have to be interpreted in the context of the relevant microenvironments within which these cells exist. However, although we are still some distance from realising these goals of molecular fingerprinting and individualised therapy for cancer, the continually expanding literature surrounding successful tumour regression with various strategies aimed at oncogene inactivation and the knowledge gained strongly suggest that the journey is worth undertaking.

## Conclusions

In several transgenic mouse models of cancer, the generation of invasive/metastatic tumours in which more than one genetic event was involved can be reversed following inactivation of a single oncogene. These findings suggest that some metastatic lesions may remain responsive to therapeutic intervention originally targeted to the primary lesion. Recent data from a mouse model of osteogenic sarcoma showed that even transient c-Myc inactivation can result in sustained tumour regression [19].

Here we show the consequences of inactivating c-Myc transiently in two distinct tumour types *in vivo*. In contrast to the osteogenic sarcoma model, re-activating c-Myc in islet β-cell tumours does not lead to accelerated β-cell apoptosis, but rather restores the oncogenic properties of c-Myc, rapidly re-initiating β-cell proliferation, loss of differentiation, loss of E-cadherin, local invasion and angiogenesis. This occurs despite the re-differentiation of previously c-Myc-activated tumour cells to a more mature phenotype and the loss of some of the newly acquired vasculature, occurring during the period of c-Myc inactivation. Similarly, in epidermis, reactivating c-Myc in suprabasal keratinocytes does not result in apoptosis, which remains confined to the shedding areas of parakeratosis at the skin surface, but restores the papillomatous phenotype, inducing cell proliferation and dysplasia.

The differences between the conditional tumour models used by ourselves and Jain et al. [19], rather than detracting from the conclusions drawn as is frequently the case, serve to highlight the importance of identifying different cellular contexts in which transient inactivation of oncogenes may provide a valid therapeutic approach. These results are significant in that they suggest that epigenetic changes resulting in increased sensitivity to apoptotic stimuli will be determining the effects of altering Myc levels. Although it remains to be seen whether transient inactivation of other oncogenes can result in sustainable tumour regression, these studies begin to define the requirements necessary for transient c-Myc inactivation to be effective as a cancer therapy. Thus, we would challenge the potential for cancer therapies aimed at transient oncogene inactivation, at least under those circumstances where tumour cell differentiation and alteration of epigenetic context fail to reinstate apoptosis and no alternative mechanism exists for tumour cell removal. One would also have to be cautious about therapies that instead of removing cancer cells might rely largely on promoting re-differentiation – such 're-differentiated' cancer cells could probably all too readily reacquire their cancer potential.

Together, these results suggest that treatment schedules will need to be informed by knowledge of the molecular basis and environmental context of any given cancer.

## Methods

### Transgenic mice

*pIns-c-MycER*^*TAM *^and *Involucrin-c-MycER*^*TAM *^mice were generated by cloning a full-length human c-*myc *cDNA fused to the hormone-binding domain of a modified estrogen receptor (c-MycER^TAM^) downstream of the rat insulin promoter and the human involucrin promoter, respectively, as previously described [8,9]. DNA constructs were injected into male pronuclei of day 1-fertilized (CBA × C57BL/6)F1 embryos and injected embryos were transferred into day 1-plugged pseudopregnant foster mice and the litters screened for presence of the transgene by Southern blotting. Heterozygous founder mice were backcrossed appropriately to establish transgenic lines.

Heterozygous *RIP7-Bcl-x*_*L *_mice were obtained from Dr Doug Hanahan [22]. Litters from all transgenic mice and appropriate F1 crosses were routinely genotyped by PCR analysis on genomic DNA (1 to 5 μl) isolated from ear biopsies. DNA was extracted by incubating each ear disc in "Hotshot" reagent (25 mM NaOH, 0.2 mM disodium EDTA; pH12) for 10 minutes at 95°C. Following this, 75 μl of neutralizing agent (40 mM Tris-HCl, pH5) was added and the sample cooled to 4°C overnight. Primers used for the detection of *c-MycER*^*TAM *^cDNA: (forward) 5' CCA AAG GTT GGC AGC CCT CAT GTC 3'; (reverse) 5' AGG GTC AAG TTG GAC AGT GTC AGA GTC 3'. PCR program: 94°C 2 min 1 cycle, [94°C 1 min, 57°C 1 min, 72°C 2 min] 30 cycles, 72°C 10 min 1 cycle. PCR product size: 413 bp. Primers used for the detection of *RIP7-Bcl-x*_*L *_cDNA: (forward) 5' AGC ACT TTC TGC AGA CCT AGC AC 3'; (reverse) 5' CAG CTC CCG GTT GCT CTG AGA C 3'. PCR program: [94°C 1 min, 60°C 30 s, 72°C 2 min] 30 cycles, 72°C 3 min 1 cycle.

Transgenic mice were housed under barrier conditions with a 12 hour light/dark cycle and access to food and water *ad libitum*.

### Activation and inactivation of c-MycER^TAM ^protein

Expression of the chimeric protein, c-MycER^TAM^, was targeted to pancreatic β-cells using a rat insulin promoter, or to suprabasal keratinocytes using the human involucrin promoter. As shown in our previous publications [8,9], the transgenically expressed c-MycER^TAM ^protein remains inactive due to association of the cells' own hsp90 with the ER^TAM^. Upon administration of 4-hydroxytamoxifen (4-OHT), hsp90 is displaced allowing association of c-Myc's partner, Max, to form transcriptionally active heterodimers [33].

To activate c-MycER^TAM ^protein in pancreatic β-cells of adult transgenic mice, 1 mg of 4-OHT (Sigma) sonicated in peanut oil (1 mg/0.2 ml) was administered daily by IP injection. To activate c-MycER^TAM ^protein in skin epidermis of adult transgenic mice, 1 mg of 4-OHT (Sigma) dissolved in ethanol (1 mg/0.2 ml) was administered daily by topical application to a shaved area of dorsal skin.

Inactivation of c-MycER^TAM ^protein was achieved following withdrawal of 4OHT. As c-MycER^TAM ^RNA and protein levels remain unchanged in the presence or absence of 4OHT, Northern and Western blot analysis will not confirm whether the protein is inactive. Thus, to confirm inactivity of the c-MycER^TAM ^protein in pancreatic β-cells, we show reversal of several markers of Myc activation by day 4 of 4OHT withdrawal -growth arrest, re-differentiation, re-establishment of cell-cell contact – by immunohistochemistry (see ref [9] and Results section). In addition, we have gene array data confirming the rapid normalisation of Myc-regulated gene expression, of tamoxifen withdrawal (eg. insulin, pdx-1, Isl-1, cyclin D; data not shown).

Similarly, inactivation of c-MycER^TAM ^protein in skin epidermis was confirmed using immunohistochemistry for markers of re-differentiation (K1 and K14) and growth arrest (see Results section). The tight regulation of the c-MycER^TAM ^protein in skin epidermis was also previously shown in [8] using *in situ *hybridisation for detection of ODC RNA, a known c-Myc target gene; by day 5 following withdrawal of 4OHT, ODC RNA is no longer detected.

### Histological and immunohistochemical analysis of pancreatic tissue

Pancreata or skin were excised from mice and 5–10 mm pieces of tissue were fixed overnight in neutral-buffered formalin, embedded in paraffin wax and sectioned (5–10 μm). Frozen sections were prepared from tissue embedded in OCT and frozen in foil on a bath of dry ice and ethanol. Prior to staining, frozen sections were air-dried and fixed in 4% paraformaldehyde for 15 minutes. Alternatively, for frozen sections, tissue was fixed in 4% paraformaldehyde for 2 hours followed by incubation in 30% sucrose overnight at 4°C. For pancreata analysis, sections (5–10μm) were cut throughout the entire pancreas and every tenth section was selected for histological and immunohistochemical examination. For skin, sections (5–10μm) were cut through two 10 mm pieces of tissue and every tenth section was selected for analysis.

Primary antibodies were as follows: rabbit polyclonals Ki-67 (Novacastra) and Nkx6.1 (Ole Madsen, NovoNordisk); rabbit anti-mouse laminin (Sigma); guinea-pig anti-porcine insulin (Dako); rat anti-mouse E-cadherin, (Zymed); rabbit anti-mouse keratin 1 (BabCo); rat anti-mouse CD45 (AbCam). E-cadherin and laminin antibodies were found to label reliably only frozen tissue sections. Other antibodies were effective when used on both paraffin-embedded and frozen sections, although Ki-67 and Nkx6.1 required epitope retrieval by microwaving paraffin-embedded sections at 700 W for 2 × 10 minutes in 0.01 M citrate buffer, pH6.0 (Vector) followed by immersion in cold water.

Antibodies were diluted in incubation buffer: PBS/0.5% Triton X-100 containing 1:25 dilution of serum from the same species as the secondary antibody. Primary antibodies for insulin and Ki-67 were applied together to sections for 1 hour. Sections were then incubated in Texas Red-conjugated goat anti-guinea pig Ig secondary antibody together with FITC-conjugated goat anti-rabbit secondary antibody (Vector). After washing, sections were mounted in Vectashield mounting medium (Vector).

To detect cells undergoing apoptosis, costaining with TUNEL/insulin, TUNEL/laminin and TUNEL/K1, immunofluorescent staining was performed by applying insulin, laminin or K1 antibodies to sections for 1 hour at room temperature followed by Texas Red-conjugated goat anti-guinea pig (for insulin antibodies) and goat anti-rabbit (for laminin and K1 antibodies) Ig secondary antibody (Vector). TUNEL staining was subsequently performed using ApopTag Fluorescein Direct kit (Chemicon) for frozen tissue sections and ApopTag Fluorescein Indirect kit (Chemicon) for paraffin-embedded tissue sections.

## Authors' contributions

SP participated in the design of the study, administered 4OHT to relevant mice, collected tissue, carried out immunohistochemical staining, coordinated and analysed data, drafted the manuscript, and provided part of the funds. SA carried out genotyping, assisted with the administering of 4OHT to mice, collected tissues, cut sections and assisted with capturing of images. LC assisted with genotyping, cutting of sections, and immunohistochemical staining. VI assisted with genotyping and immunohistochemical staining. SZ assisted with genotyping and immunohistochemical staining. MK participated in the design of the study, assisted with the coordination and analyses of data, helped draft the manuscript and provided funding.
